# Dignity in people with frontotemporal dementia and similar disorders — a qualitative study of the perspective of family caregivers

**DOI:** 10.1186/s12913-017-2378-x

**Published:** 2017-06-23

**Authors:** Mette Sagbakken, Dagfinn Nåden, Ingun Ulstein, Kari Kvaal, Birgitta Langhammer, May-Karin Rognstad

**Affiliations:** 1 Department of Nursing and Health Promotion, Faculty of Health Sciences, Oslo and Akershus University College, Oslo, Norway; 2 The Norwegian Centre for Migration and Minority Health (NAKMI), Oslo, Norway; 3 Department of Psychiatry of Old Age, Oslo University Hospital, Ullevaal, Oslo & Institute of Clinical Medicine, University of Oslo, Oslo, Norway; 4 Inland Norway University of Applied Sciences, Faculty of Public Health, Institute of Health Sciences, PBox 400, N-2418 Elverum, Norway; 5 Department of Physiotherapy, Oslo and Akershus University College, Faculty of Health, Pb 4, St Olavs pl, N-0130 Oslo, Norway

**Keywords:** Dementia, Dignity, Relatives, Nursing homes, Day care centre

## Abstract

**Background:**

Frontotemporal dementia (FTD) constitutes on average 10–15% of dementia in younger persons (≤65 years old), but can also affect older people. These patients demonstrate a decline in social conduct, and/or language aphasias, apathy, and loss of insight that is gradual and progressive. Preservation of dignity seems to be highly relevant both before and after admission to different types of institutionalized care, but the research is scant. From the perspective of close relatives, this study aims to develop knowledge related to dignified or undignified care of patients with FTD and similar conditions.

**Methods:**

A qualitative, descriptive, and explorative design were employed to address the aims of this study. We interviewed nine relatives of people with FTD and similar conditions living in nursing homes, and two relatives of people living at home but attending day center 5 days a week.

**Results:**

Relatives described the transition from being a close relative to someone who had little influence or knowledge of what constituted the care and the daily life of their loved ones. According to relatives’ descriptions, patients are deprived of dignity in various ways: through limited interaction with peers and close relatives, limited confirmation of identity through staff who know them well, lack of possibilities for making autonomous decisions or entertaining meaningful roles or activities. Examples provided from the day care centres show how dignity is maintained through identity-strengthening activities conducted in different places, under various kinds of supervision and care, and together with people representing different roles and functions.

**Conclusions:**

Maintaining a link with the world outside the institution, through closer cooperation between the institution and family members, and/or by the use of day care centres, seems to facilitate prevention of many of the factors that may threaten dignified care.

## Background

Frontotemporal dementia (FTD) is a pathologically and clinically heterogeneous group of non-Alzheimer neurodegenerative dementias [1]. People with neuronal degeneration in frontal and temporal lobes demonstrate a decline in social conduct, and/or language aphasias, apathy, and loss of insight that is gradual and progressive [1, 2]. These symptoms can overlap with a range of other conditions, such as a frontal variant of Alzheimer’s disease or atypical parkinsonian disorders, and the diagnostic process is considered complex [1]. Earlier studies show that FTD constitutes 10–20% of dementia in younger persons (≤65 years old) [3, 4], but it can also affect older people. A recent systematic review shows that population-based estimates for the prevalence of FTD vary widely. According to this review, FTD accounted for an average of 2.7% (range 0–9.1) of all dementia cases in prevalence studies that included persons aged 65 and older, compared to an average of 10. 2% (range 2.8–15.7) in studies including those aged less than 65 years [1].

Family members often experience guilt and shame because of the behaviour of the patient when taken care of at home, and various behavioural problems cause great challenges to family caregivers and to staff after admission to different types of institutionalized care [5, 6]. Preservation of dignity therefore seems to be highly relevant both before and after the affected person receive care outside the home.

From the perspective of close relatives, this study aims to develop knowledge related to dignified or undignified care of patients with FTD and similar conditions living in nursing homes or using associated day care centres. This paper is part of a comprehensive study that comprised interviews with relatives including topics related to views and experiences both in the period before and after receiving help from outside of the home. The study also comprised interviews with health personnel exploring environmental factors and activities potentially set in place to ensure dignified care. In addition, an intervention study based on the findings from all participants, is currently being conducted.

This particular paper focuses on the views of family members concerning deprivation of dignity, but also illuminate factors that help preserve patients’ dignity.

### On dignity

The theoretical perspectives of dignity which are employed in this study are from the Nordic scholars Katie Eriksson [7, 8], Margareta Edlund [9] and Lennart Nordenfelt [10, 11], respectively representing a perspective of caring science and a philosophical perspective on medicine and healthcare.

The human being is viewed as having two forms of dignity: absolute and relative dignity. Absolute dignity refers to human beings having, by virtue of being created, an inalienable dignity. Relative dignity is influenced by culture and society, and can have different connotations in different contexts. It can be transformed, torn down, and rebuilt [7, 9, 12]. Nordenfeldt [10] presents four kinds of dignity. Dignity of moral stature, which is the result of the moral deeds of the person, is dependent on the public image of a person’s dignity of moral stature, and of the thoughts and deeds of the subject. According to Nordenfeldt, this kind of dignity is tied to actions of exceptional moral value. In our view, this understanding of dignity does not imply that persons with frontotemporal dementia do not possess dignity of moral stature, but it may be blurred or invisible because of the person’s illness and his/her uninhibited behaviour. Dignity of merit depends on social rank and position, and where certain rights are attached to the position. This kind of dignity is unevenly distributed among human beings, it exists in degrees, and it can come and go [10].

The dignity of identity is concerned with the integrity of the subject’s body and mind, and it may also depend on the subject’s self-image. The dignity of identity can come and go as a result of the actions of fellow human beings or as a result of changes in the subject’s body and mind. Persons living with dementia are often experiencing an ongoing loss of personal dignity, value and security, and experiencing cognitive decline can be a threat towards self-respect and the sense of dignity [13]. Nordenfelt [10] considers identity to be one of the most important issues in the contexts of illness and ageing, because of “our attachment to ourselves as integrated and autonomous persons, with a history and a future, with all our relationships to other human beings” (p.33). Thus, a human beings’ dignity involves the right to be confirmed as a unique human being, with individual needs and preferences [8, 14]. Lastly, universal human dignity, or *Menschenwürde,* pertains to all human beings to the same extent and, according to Nordenfelt [10], it cannot be lost as long as the persons exist. We consider that dignity of identity in particular is essential for this present study.

### Previous empirical research

We did not find studies on dignity, within the frames of care homes, which included both patients with FTD (or dementia with similar behavioural symptoms) and their families. The majority of studies regarding these patient groups have focused on the burden associated with caregiving. Further, most research within this area focuses on being a family caregiver of persons with diagnoses of dementia other than FTD living in nursing homes.

Rosness et al. [4] found that caregivers of patients with FTD were significantly less satisfied with the provision of information about the disease, counselling, and follow-up concerning how to manage the situation compared with caregivers of patients with early onset Alzheimer’s disease. De Vugt et al. [15] found in a study on the impact of behavioural problems that spousal caregivers of persons with FTD reported higher levels of general burden and felt less competent than caregivers of those with Alzheimer’s disease. Tornatore and Grant [16] studied the satisfaction of family caregivers with nursing homes after placement of a relative with Alzheimer’s disease or a related dementia. The authors concluded that if family satisfaction is to be achieved, the relatives need to feel a positive involvement in, and influence over, the care of their relative. Riedijk et al. [17] concludes that caregivers in general need extensive support in coping with the situation of their family member with FTD.

In a study exploring the role of relatives in relation to persons with dementia living in special care units in Norwegian nursing homes, it was found that as long as the resident expressed satisfaction and the relatives perceived that everything was fine, their behaviour was rather passive. However, when the relatives perceived changes in the situation and a reduction in the resident’s well-being, they took on a more active role. Thus, the relatives did not hold a stable role or function in their family member’s daily life, but rather moved between different roles containing different degrees and ways of participation, depending on what they considered that the resident at any time were lacking [18].

Sury et al. [19] reviewed the literature and found that successful transitions to nursing homes may be assisted by orientation procedures for the person with dementia and family members prior to the admission, a “buddy” system for new arrivals, and a person-centered approach. According to Johansson et al. [20] negative expectations of dementia care make separation more difficult in the process of relinquishing the care of a person with dementia to a nursing home. Having family caregivers recognized as partners in the care of the person with dementia can facilitate adaptation to the new situation in the nursing home [21]. Identity cues seem to play a central role in communication and constitute important information that the family caregivers can share with health care personnel with regard to a patient living with dementia [22].

Only one study, which focuses on dignity in persons with dementia living in nursing homes from the perspective of family caregivers, was identified. Heggestad et al. [23] found that the most important issue from the perspective of family caregivers was that their family member with dementia living in a nursing home should receive confirmation as a relational human being.

### Aim of the study

Based on our review of the literature, we found it important to focus on dignity and indignity in persons with FTD and similar conditions, which has apparently received only scant attention. This study, aims to develop knowledge related to dignified or undignified care of patients with FTD and similar conditions from the perspective of close relatives.

## Methods

### Study design

We employed a qualitative, descriptive, and explorative design to address the objective of this study. The descriptive part reflects the direct exploration, analysis, and description of how relatives experience the way that dignified care is ensured or neglected in the context of a nursing home and in the context of a day care centre. The explorative part reflects the aim of gaining new insights into the experiences of relatives from the relatively scant knowledge in this field, thus discovering new ideas, and increasing knowledge [24]. Examples provided from relatives related to the day care centers were used as a counter-point to those with a relative living in residential care.

### Study setting and study population

We included four nursing homes with special care units for patients with dementia in the city of Oslo and the surrounding county of Akershus in this study. The special care units were located in ordinary nursing homes but are adjusted for dementia care in the sense that the units are small, the number of patients few, and the staff should hold high and relevant competence. To protect patients from sensory overload the units are sparsely furnished and the patients’ rooms contain few personal items like ornaments and pictures. Many nursing homes in Norway have associated day care centres (located in the same building). The day care centres can be for all types of elderly patients or for patients with dementia in particular. In two of the families, the person with dementia attended a day care centre for people with dementia 5 days a week. The remaining nine patients were permanent residents in special care units in the nursing homes. The head nurses recruited relatives of patients who were admitted to the nursing home and who represented the described patient group. A psychologist employed at one of the nursing homes recruited the participants that had relatives attending the associated day care centre. Seven patients had received a diagnosis of FTD, and four patients had other dementia types that included pronounced behavioural disturbances. The patients were in the age range of 57–90 years. Eleven relatives, nine women and two men, were recruited to participate in individual interviews. The participants were between 46 and 78 years and consisted of five spouses, four daughters, one sister, and one nephew. They all represented close relatives in the sense of being primary caregiver before admission. After 11 interviews, we reached saturation in the sense that we experienced extensive repetition of the relatives’ descriptions of their experiences related to dignified and undignified care.

### Data collection

We conducted 11 semi-structured interviews lasting between 45 and 60 min. The semi-structured interview guide derived from the research questions and was inspired by theoretical accounts of the concept of dignity and empirical studies. However, the approach was flexible in the sense that the interviews were partly governed by answers and topics introduced by the informants. Some of the themes related to the period before diagnosis, and when the family member was still living at home. These findings are to be published elsewhere.

**Table:** 

Key topics covered by the interview guide
- Situation at home/work when the family member started to show symptoms of dementia - The diagnostic process - Situation of family caregiver when the family member stayed at home - Admission to suitable care - Cooperation with staff working in nursing homes/day care centres - Environmental factors - Daily life activities of patients in nursing home/day care centres - Maintenance of dignity when aggression /behavioural disturbance was managed on the ward - Examples of situations of good care/preservation of dignity - Examples of situations of poor care/violation of dignity

The overall aim of the interview was to gather information about dignified and undignified care, seen from the perspective of the relative, and to interpret the meaning of these descriptions [24]. The relatives chose the time and location for participation in the interviews. Two relatives were interviewed in the nursing home where their family member lived. Nine relatives preferred to be interviewed in a neutral office room at Oslo and Akershus University College, which is conveniently located in the centre of Oslo. All interviews were tape recorded and transcribed verbatim.

### Ethical issues

The Health Research Act and the guide to the Act provided by the Ministry of Health and Care Services (English translation) define what falls within and outside the concept of “medical and health research” in Norway. Approval for this study was not needed from the Regional Committees for Medical and Health Research Ethics in Norway. Quality assurance projects and research that uses personal data, which falls outside the scope of the Health Research Act, are to be reported to the Norwegian Social Science Data Services (NSD). This study was reported to NSD and granted permission before the data collection started.

Participants received oral information and a written informed consent form from the head nurse or the psychologist assisting with the recruitment of participants. All the relatives gave permission to be contacted by the interviewer. During the first meeting with the participant, the interviewer repeated the information about the study. The participants were informed that their participation was voluntary and that they had the right to terminate their participation without giving a reason. All participants signed the informed consent form.

The findings of this study, including findings that comprised examples of undignified care, are to be brought back to the different nursing homes in the form of a lecture and a short report. One of the nursing homes is already involved in an intervention study based on the results of the study. If any undignified care, that constituted abuse, had been uncovered (which was not the case), the incident would have been reported to the head of the department. If the head of the department had not taken necessary measures, the incident would have been reported to the Norwegian Board of Health.

### Analysis and interpretation of data

Our research method is inspired both by phenomenology and hermeneutics, and represents a combination of both; a phenomenological hermeneutical approach [25]. According to Kvale [24], phenomenology focuses on the human experience while hermeneutics is the theory and methodology of text interpretation. As the interviews provided expressions related to relatives’ perceptions and experiences in regards to the care of patients with frontotemporal dementia and similar conditions, we intended to explore the essential meaning of their descriptions. We adopted Kvale’s [24] interpretation as he argues that one should strive to understand phenomena as they are experienced and described by the participants of the study. The interviews with the relatives were semi-structured, and were tape-recorded and written down, producing text that could be interpreted. The analytical method focused on the meaning of the participants lived experiences, interpreted through a dialectical process involving interpretation of the produced text [24]. Kvale’s [24] three levels of interpretation were used as a strategy to help structure the interpretations: interpretation at the self-understanding level, the common-sense level, and the theory level. At the self-understanding level, the researchers formulated, in a condensed form, what the subjects themselves understood to be the meaning of their statements [24]. This first step involved three of the co-writers reading the transcribed interviews several times in their entirety to acquire an overall impression of the content. The next part of this process involved searching the entire material for similar and contrasting statements. Units of meaning, representing factors associated with dignified and undignified care, were identified by color-coding in order to structure the participants’ utterances in the texts. After several discussions, related to which units of meaning represented dignified and undignified care, meaning condensation was conducted. The condensed text thus represented a rephrased condensation of the meaning of the interviewees ‘statements from their own viewpoints, as understood by the researcher.

The next step, which involved a critical common-sense understanding, went beyond reformulating the subjects’ self-understanding and included a wider frame of interpretation. At this level, further attentive reading and discussions uncovered nuanced meanings related to the initial meaning units. By adding general knowledge about the content of the statement, we made it possible to amplify and enrich the interpretation of the participants’ statements [24]. Thus, this part of the analysis moved from units of meaning and generated preliminary themes by labelling the short sentences with sub-themes to structure the text further.

In the last phase, the different sub-themes were linked together and described in central themes that reflected the focus of the study [24]. This more comprehensive interpretation involved contextualizing the critical commonsense understanding by using theoretical frameworks and previous research moving our analysis to a higher level of abstraction. The third context of interpretation is reflected in the discussion. The frameworks that represent other theoretical perspectives than dignity were included as a response to findings emerging from the field.

In Table 1, we show examples of how units of meaning, identified in the text via coding, were condensed, and how subthemes and central themes were identified and described.

**Table 1:** Example of themes and structure

Unit of meaning	Condensation	Sub-theme	Central theme
“I do not get any information about activities he participates in. But then you discover that he has been participating in something, but you don’t get any notice beforehand. They could make a plan over expected activities and inform the relatives that they were welcome to participate” “Other patients are coming into her room, laying down in her bed, and they are coming in … in to use her toilet … and things like that … that has nothing to do with the personnel … but it has to do with the whole situation … making her really angry and frustrated”	Information about ongoing activities for the patients is lacking. An activity plan, that involves relatives, is requested. Other patients enter the room, using the bed and toilet. Being deprived of privacy causes anger and frustration.	The relatives are not familiar with the department’s activities and daily routines. The relatives would like to participate in activities. Challenging to organize care that implies respecting and safeguarding patients with different needs. Those that function better than others may feel humiliated.	Involvement of relatives Undignified life situation

## Results

Central findings related to dimensions of dignified and undignified care are reflections of the perceptions of the family caregiver on behalf of the person living with dementia. Some of the perspectives were gathered through direct questions about dignified and undignified care. However, many of the answers were reflections on issues that describe undignified care, without the respondents’ using the concept of dignity. Subsequently, the themes described below are identified through both direct and indirect questions and answers about dignity in care.

### Preservation of roles and activities

Even though the questions were centred on dignity related to the person living with dementia, one of the central recurring themes raised by the family caregivers was related to the lack of involvement of the relatives. Furthermore, many of the subthemes link to this particular theme. Relatives were most of the time only included as resources and dialogue partners during the process of admission to the nursing home. After the admission, relatives often felt excluded and had little knowledge of what constituted the care and daily life of their loved ones. The transition from being a family caregiver with daily responsibilities to being someone seldom consulted or informed was described as difficult. A daughter responds to a question related to whether she in any way had been used to adjust the care for her mother:

“Not more than what we … the form we filled out in the beginning. With all the names and addresses, interests and background, siblings and so on […] but nothing after that really.”

There was a tendency throughout almost all the interviews for relatives to inquire about what they called “everyday life information”. Many had tried to establish a regular dialogue by asking for regular meetings or telephone calls, or encouraging the health personnel to write in diaries placed in their family member’s room. The absence of a system with primary nurses made this difficult, and communication between health personnel and relatives became sparse and fragmentary. A relative tells how she keeps on meeting new nurses without any of them being someone she can have a continuous dialogue with:

“There are many people that we see, that show up and that we meet … without them being … (primary nurses). I don’t have any knowledge of them.”

Relatives described that they felt alienated and insignificant in a context where it was difficult to access and communicate with someone who knew their family member well. Many spoke of how they felt neglected — not being told about, or invited to, social activities in the nursing home, or invited to participate in meals or other ordinary situations during the day. Some described the visits as artificial and odd, with the relative striving to find things to do or talk about. The rooms’ being stripped of personal items, like paintings, pictures and ornaments that facilitate talks, seemed to be inhibiting. A relative tells how they keep repeating the same activities during visits:

“We sit and talk to her, yes … and then we go through the mail. […] Then we check that everything is OK … her income tax form when it comes … and the like … and then we organize her wardrobe and clothes, and check that everything is in order.”

Relatives seemed to be trapped in the rules and routines of the department; only very few managed to initiate activities beyond passively engaging with their family member in his/her room. Some relatives suggested that having the possibility of being included in ordinary activities like cooking or meals could have resolved some of this. A few relatives did manage to find ways to ensure meaningful activities by taking their family member out of the nursing home, or by managing to conduct activities within the frames of the special care unit. A relative explains how she found ways to ensure that her father was given what she called a dignified life:

“We would make some food, sitting down having lunch together. I would buy shrimps, and he would peel the shrimps himself. When the nursing home did not manage to reach him in his particular situation, then I had to … I felt that it was important that I contributed to ensure that he got a dignified life … including hiring this private nurse for him.”

Even though some relatives managed to find ways to ensure personal and dignified care, the characteristics of the patient group and the structure of the departments seemed to create structural or mental barriers that were difficult for the relatives to infringe upon. The findings show an exception among those who used the day care centre, even though these participants also had family members with uninhibited and sometimes aggressive behaviour. The context of the day care centre seemed to allow for less strict routines and rules, combined with a wider range of activities. A spouse responds to a question on whether her husband participates in activities in the day care centre:

*“Yes he does… He…he has become so much better after he started to go there (day care centre). It works very well. He is calm…and he talks more…he is engaging […] In many ways, he now seem so resourceful…”*

The relatives of those who used the day care centre also reported closer contact and continuity in the dialogue between the health personnel, patient, and relative — making it possible to play an active part in shaping daily activities as well as to maintain knowledge of the family members’ daily life. The contact constituted regular meetings, telephone calls, as well as regular interaction due to the relatives frequently delivering or collecting their family member or participating in activities in the day care centre.

### Individual confirmation and influence

During the interviews, dignified care was linked with personalized care. Many participants asked for activities associated with previous or present interests, and several gave examples of possible activities’ being overlooked, such as engaging patients who had formal or informal skills or interests (for example, in gardening, cooking, and carpentry) in relevant daily activities. This type of individual stimulation was associated with a sense of purpose that could reduce feelings of isolation and alienation. A relative describes being uneasy about his relatively young wife being offered the same types of activities as older patients:

“The opportunities that they are offered (activities) are not adjusted for patients like her. It is these “memory moments”, and entertainment with some strange musicians, and … and sit-down training … and God knows what. It doesn’t make sense, she is still functioning adequately mentally, and she has had relatives visiting her […] and they are shocked to see her there.”

Another theme related to personalized care that arose was allowing patients to decide on, and to influence, their own care. Even though the capacity to make autonomous decisions varied, many found it undignified that there were so few opportunities for their family members to make their own decisions. A relative described how she found it hard that her father did not have the opportunity to request what he liked for breakfast:

“Father wants cornflakes in the morning, but the nursing home does not want to buy cornflakes because there are only three residents in the nursing homes who have asked for cornflakes.”

Some participants, in particular those who had their family members at the day care centres, provided examples of more person-centred care. These participants described how daily activities both seemed adapted to their family members’ skills and preferences and were negotiable in accordance to daily needs. A wife relates how her husband was offered various types of activities at the day care centre, but at the same time how his present needs were decisive with regard to participation at that particular moment:

“They often try to take him out walking, but sometimes he does not want to […] and then he stays home (at the centre). […] They try to engage him, as when they told me that they had received some new furniture and he sat there and worked and screwed it together, and after a while he took over and completed the whole thing.”

Dignified treatment was also associated with individual confirmation in the sense of proper versus inadequate personal care. Lack of individualized hair care, disappearance of clothes, family members’ wearing other residents’ clothes, and combinations of clothes seen as unsavoury were described as examples of undignified care. Lack of individual care was associated not only with negligence but also with relatives’ not playing any part in organizing the care.

### Living in a “closed system”

A majority of the relatives brought up their insecurity related to what characterized patients living in the special care unit, and the rationale behind their family member’s being placed in such a unit. Many seemed to think that their relative was different from the others and described feelings of humiliation on behalf of their family members because of their having to interact with persons who behaved in a way that often demanded active seclusion, physical restraint and extensive follow-up. A relative describes how she perceives her father’s situation:

“It sounds like he is angry, because he does not meet any at the same age or who he can talk to … and we have reacted to this too. We are thinking ‘OK, he is in a special care unit (skjermet enhet), and maybe it is like this when you are in a special care unit’.”

A theme that often triggered positive responses was related to the ability of health personnel to preserve patients’ dignity during situations where active seclusion, physical restraint or other types of milieu therapeutic strategies were needed. One relative reported that her father was “treated with love” in such situations and that she felt “touched” because of the patience and care expressed by the health personnel. However, because of the frequency of indiscriminate or aggressive behaviour among some of the patients, relatives suggested that health personnel should “compose” groups of patients that would fit better together and subsequently facilitate activities and conversations between persons at similar levels of functioning.

By the same rationale, many questioned the need for isolating their family members to the degree that was practised and found it difficult to accept that the doors in the special care unit were locked. One daughter described how her mother, who used to love going for long walks, had become so worried about not being able to re-enter the nursing home that she had become hesitant to go for walks with her. Another relative emphasized the effect of the institutional system, saying “the system does not make allowances for these types of patients”, while others used phrases like living in “a closed system” or “a closed box” to describe how the physical space and the general rules of the special care unit affected the individual. A relative describes her feelings the first time she left her mother:

“When I entered the nursing home and saw that small room, the room being naked and almost sterile. […] I thought that now we are delivering her into a … into a deposit box, and technically it was similar to her being dead.”

Many worried that their family members did not have the possibility of going out, or were left alone too much, some relating this to the allocation of too many resources used to prevent aggressive or indiscriminate behaviour among certain patients. A relative expresses her concern:

“If I am to say what I mean, he is left alone far too much because of the others (patients) demanding so much attention, taking so much time … and he cannot stand to be together with these nagging persons. […] So my dream is that he could be in a place that was more in line with his level … but that still had the resources.”

The quote summarizes what seem to be conflicting needs of many in this patient group: several are relatively young and physically fit, with adequate levels of expression, but still in need of a special care unit because of isolated or frequent events of aggression or lack of impulse control.

## Discussion

Central findings related to the dimensions of dignified and undignified care included the lack of involvement of relatives. Relatives also expressed insecurity related to what characterized patients living in the special care unit, and the rationale behind their family members being placed in such a unit. According to relatives’ descriptions, their family members were deprived of dignity in various ways: through limited interaction with peers and close relatives, limited interaction with staff that know them well, lack of possibilities to make autonomous decisions or entertain meaningful roles or activities. Examples provided from the day care centres show how dignity could be maintained through identity-strengthening activities conducted in different places, under various kinds of supervision and care, and together with people representing different roles and functions.

### Dignity related to continuation of roles and relationships

One of the main finding in this study was reflected in what relatives described as loss of a meaningful role. Relatives described a transition from being a close relative to being someone who felt excluded and had little influence or knowledge of what constituted the care and the daily life of their loved ones. Several studies focus on different aspects of dignity in care of patients with dementia [26], but surprisingly few seem to integrate the aspect of preserving continuity in the relationship with the relatives. Review articles addressing good quality and dignified care for elderly people in institutions (with or without dementia) often underline the importance of person-centred care [26–28]; a philosophical approach to service delivery with an emphasis on preserving autonomy and integrity, shared decision-making, personalization of the person’s care, and the like [29]. However, many of these studies focus on the patterns of interaction between health personnel and patients rather than on integration and cooperation with relatives. In general, little attention is paid to the role of the family caregiver after nursing home placement. However, the findings in the present study do show that one of the relatives’ main concerns, emerging in a discussion about preservation of dignity, is related to the integration and maintenance of their own role as caregivers. These descriptions also seem to reflect reciprocal benefits for family caregivers in terms of maintaining their own identity, as family members and caregivers, by continuing to play a role in providing care for close family members. Based on this study, it seems to be important for all parties to acknowledge that person-centred care should include and integrate relatives as caregivers and dialogue partners.

In a study from UK, relatives who experienced nursing home placement ascribed important aspects to their role [30]. One of them was maintaining continuity, implying helping the older person to maintain his/her sense of identity through the continuation of a loving family relationship and through helping the staff to get to know the person in care. Another important aspect was monitoring the care, and filling in the gaps by providing personal and intimate knowledge about the older person. An “engaged involvement” was perceived as a way to reduce role loss and to create new ways of caring [30]. Even though this particular study did not focus on patients with dementia, it supports what appears to be an important finding about role loss in our study. Relatives described how they felt alienated and insignificant in a context where it was difficult to access and communicate with someone who knew their family member well. Many described how they made active efforts to ensure regular communication but felt neglected in the sense that efforts were not responded to and that they were not told about, or invited to, social or care-related activities in the institution. Lack of knowledge of what constituted “everyday life activities” also inhibited the interaction and small talk between the relative and the person living with dementia, all in stark contrast to the relative’s previous role as the main caregiver.

Also other studies concerning dementia and long term care have found that relatives take the initiative and responsibility to establish a relationship with the staff; that there appears to be little willingness to negotiate the nature and extent of relatives’ involvement, and that the relatives themselves must seek information actively, often without any staff being identified as in charge of the patient [31, 32]. In a study from Australia, relatives with experience of long-term dementia care placement did not feel “embraced” within the culture of the nursing home. This included not being encouraged to be involved in the care of the person living with dementia, even though the relatives expressed that they wanted to continue to play an important part in the patient’s life [32]. A study from Norway, also focusing on relatives of persons living with dementia [33], found that through the narratives provided by relatives, nursing care personnel acquired a far more nuanced picture of the person and his/her engagement in life. These narratives were essential in helping persons living with dementia to keep up with meaningful activities and to enhance their quality of life. Furthermore, the authors emphasized that identifying activities that connect the person with dementia to people in their family might help to strengthen the identity of that person. The relational context allows for a different type of mutual reciprocity — and through activities that previously were meaningful, and through genuine conversations [33], the relative might invite the person into a caring relation where the person is confirmed as a human being [34].

### Dignity related to confirmation of identity

There is a close relationship between dignity, autonomy, and integrity [10]. Being deprived of roles, the ability to influence decisions or the possibility to participate in meaningful activities can therefore be understood in the light of what Nordenfelt [10] calls “dignity of identity”; the dignity that we attach to ourselves as integrated persons including our life story, our autonomy, and our social relations. The findings in the present study indicate that patients may be deprived of identity in different ways; through limited interaction with peers and close relatives, limited confirmation of identity through staff that know them well, lack of possibilities to make autonomous decisions, lack of possibilities to entertain previous meaningful roles or activities, and lack of attention towards individual appearance. From a dignity perspective, not being seen as an individual person, or being treated as a member of a group or as someone with a certain diagnosis, may represent a threat to the identity and become what Goffman [35] calls a “total identity”. An illness or caring situation fostering such a master status will have consequences for a person’s identity and therefore for his/her dignity [11].

Preservation of some degree of freedom and some degree of autonomy is, according to Nordenfelt [10], an important part of maintaining what constitutes “the dignity of the identity”. The present findings show that even though the capacity to make autonomous decisions varied, many relatives found it undignified that there seemed to be so few opportunities for their family members to make their own decisions or to continue the lifestyle or activities to which they had become accustomed over the years. These findings are supported in another study from Norway [33], where relatives expressed their concerns related to the inability of the care recipients in the nursing homes to promote their wishes and preferences for activities and everyday life habits. Such inability was related to problems with communication because of cognitive impairment, but also to the elderly being unaccustomed to expressing their wishes to a third party (health personnel) representing public authority and not being a caregiver in the sense of being close. The inability was also related to institutional regulations being applied strictly, limiting persons with dementia with regard to exercising any autonomy in their everyday life [33]. In another study from Norway, including participant observation and in-depth interviews with long-term care residents in four nursing homes, quality of care was linked to the ability of the nursing homes to create a ‘home’ for the residents, and subsequently also to what extent health workers managed to respond to each residents’ unique needs and expectations [36].

What is required in terms of preservation of autonomy and freedom differs from person to person, and from situation to situation [10]. In the case of the patient group included in the present study, choices made in relation to autonomy and level of freedom may differ from day to day and be dependent on particular situations. However, even if people surrender some degree of autonomy in exchange for “membership” (patient with dementia) in a community (institutionalized dementia care), parts of their individual autonomy will always remain [37]. As illustrated through the examples from the residents in the day care centre, this part can be stimulated, even down to the level of allowing someone to choose cornflakes for breakfast. A practice where patients’ autonomy is neglected in situations where parts could have been preserved is an example of how residual autonomy is left out of the equation and is subsequently a violation of the dignity of the identity [11].

### Dignity related to loss of freedom

Preservation of some degree of freedom is, according to Nordenfelt [11], an important part of maintaining what constitutes “the dignity of the identity”. The present findings show how the relatives brought up their insecurity related to what characterized the patients living together, and the rationale behind their family member’s being placed in a special care unit. The findings indicate that there was a lack of information about the purpose of the unit, the physical design of the unit and the (bare) rooms, why doors were locked, and why behaviours and activities were strictly regulated. The relatives thus need to be informed that the organization of the ward has a therapeutic rationale, as patients with dementia are vulnerable to receiving too many stimuli. Many seemed to think that their family member had different needs than the majority and that they were different from the others. These statements indicate a disapproval of the subtle categorization and the associated restrictions that seemed to unite the patient group. Uneasiness related to the frames of care may be of particular relevance with regard to patients with FTD because these patients are often quite young [1]. In a study on how relatives of people with frontotemporal dementia experience the illness, all of the participants found that they needed to be assertive to gain access to what they considered appropriate services. Their relatives were often offered care in old-age dementia facilities and, without exception; the participants expressed the view that due to young age these were not appropriate [6].

Relatives seemed to be physically and mentally trapped in the rules and routines of the special care unit, and many described the visits as restrictive, artificial, and odd. Some suggested more freedom, on behalf of themselves as relatives, to move around and partake in activities like cooking, to avoid the feeling of such restriction. Such activities could, in line with Goffman [35], represent a way of counteracting alienation and total institutionalization. Goffman [35] coined the term “total institution” and related this concept to different institutions in the Western society. The “encompassing” character of these institutions, according to Goffman [35], is symbolized through the barriers to social intercourse; partly because of how institutions are constructed (locked doors, bare rooms) but also because of factors such as the way that the inner organizational life restricts interaction between people. One of the basic social arrangements in a modern society is that we tend to sleep, play, and work in separate locations under different authorities and without a strict, overall rational plan. Living a life in a special care unit for persons with dementia implies that these divisions are broken down —with a limited set of predefined activities conducted in the same place, under the same supervision, in a certain order, together with a group of people with certain characteristics [35]. The examples provided from the day care centres, on the other hand, show how these divisions are partly maintained through activities conducted in different places, under various supervision and care, and together with persons representing different roles and functions. Also in a study conducted in Sweden, it was found that a perspective of human dignity characterized the day care unit, and reflection and experiences pertaining to the individual person with dementia could be developed [38]. These findings suggests that activities conducted in different places, together with family caregivers as well as health personnel, helps to maintain a link with the world outside the institution and that such a link facilitates prevention of many of the factors that may threaten dignified care.

“Medicalization” of dementia, through different control measures, aiming at reducing the risk of aggressive or other types of indiscriminate behaviour, does not consider the ways in which the caregiving relationship and the conditions of the caregiving context affect the person living with dementia and his/her caregivers [39]. According to Bruce et al. [40], there is “a tendency for an inverse care law to apply, whereby those with the most complex problems are least likely to have their broader needs recognized and met”. Persons with frontotemporal dementia and similar conditions do represent a group with challenging and complex needs. Subsequently, there is a risk that their broader needs, including the need for preservation of some degree of autonomy and integrity, some degree of freedom, and preservation of continuity in their relationships with their primary caregivers, are neglected. Values, which in one way or another give expression to the dignity of identity, can in different ways be subjected to violation leading to the experience of loss of dignity [7, 9].

### Strengths and weaknesses

Our study is based on in-depth interviews with relatives of patients with FTD and similar conditions, either admitted to nursing homes or users of day care centres. Methodological rigour in the study was sought by continuous discussions with all the co-authors during the whole research process; all authors meeting at regular points for discussion of the study design, data collection and the overall interpretation and presentation of the material. The co-authors represent a varied educational background (nursing, medicine, physiotherapy), as well as a varied clinical experience, and subsequently many frames of references have come into play. Three of the authors independently read all the transcripts from the interviews before a coding frame was agreed upon. The same three authors continued a detailed and thorough analytical discussion in regards to the development of sub-themes and themes that were to constitute the main findings of the study; all in all a process that has increased the validity of the study. We included four different nursing homes with special care units for patients with dementia in the city of Oslo and the surrounding county of Akershus in this study. Even though the sample is small, we consider that the strength of the study lies in the homogeneity and coherence in the answers between different groups of relatives.

## Conclusion

The majority of the relatives felt that their family member was different from the other patients in the ward with respect to age, symptoms, functioning and needs. Many did not understand the reason why their family member was in a special care unit, and the rationale behind various care strategies. Thus, the study shows the importance of providing information to the family caregivers about the disorder, including the rationale behind different types of care and milieu therapeutic strategies. The staff may also need to strengthen their competence or awareness in regards to how to handle this demanding patient group in a respectful way. Investing in a close and continuous dialogue with the family caregivers seems particular important, which can be achieved by showing interest in their views and requesting information about the person’s premorbid function as well as her/his likes and dislikes. Maintaining a link with the world outside the institution, through closer cooperation between the institution and family members, and/or by the use of day care centres, seems to facilitate prevention of many of the factors that may threaten dignified care.

## Abbreviations

FTD: Frontotemporal dementia
